# A comparison of semi-automated volumetric vs linear measurement of small vestibular schwannomas

**DOI:** 10.1007/s00405-018-4865-z

**Published:** 2018-01-15

**Authors:** Samuel MacKeith, Tilak Das, Martin Graves, Andrew Patterson, Neil Donnelly, Richard Mannion, Patrick Axon, James Tysome

**Affiliations:** 1 Cambridge Skull Base Unit, Department of ENT, Addenbrookes Hospital, Cambridge University Hospitals, Cambridge, CB2 0QQ UK; 2 Department of Neuroradiology, Addenbrookes Hospital, Cambridge, UK; 3 Department of Radiology, Addenbrookes Hospital, Cambridge, UK; 4 Department of Neurosurgery, Addenbrookes Hospital, Cambridge, UK

**Keywords:** Vestibular schwannoma, Acoustic neuroma, Measurement, Volumetric, Semi-automated

## Abstract

**Objective:**

Accurate and precise measurement of vestibular schwannoma (VS) size is key to clinical management decisions. Linear measurements are used in routine clinical practice but are prone to measurement error. This study aims to compare a semi-automated volume segmentation tool against standard linear method for measuring small VS. This study also examines whether oblique tumour orientation can contribute to linear measurement error.

**Study design:**

Experimental comparison of observer agreement using two measurement techniques.

**Setting:**

Tertiary skull base unit.

**Participants:**

Twenty-four patients with unilateral sporadic small (< 15 mm maximum intracranial dimension) VS imaged with 1 mm-thickness T1-weighted Gadolinium enhanced MRI.

**Main outcome measures:**

(1) Intra and inter-observer intraclass correlation coefficients (ICC), repeatability coefficients (RC), and relative smallest detectable difference (%SDD). (2) Mean change in maximum linear dimension following reformatting to correct for oblique orientation of VS.

**Results:**

Intra-observer ICC was higher for semi-automated volumetric when compared with linear measurements, 0.998 (95% CI 0.994–0.999) vs 0.936 (95% CI 0.856–0.972), *p* < 0.0001. Inter-observer ICC was also higher for volumetric vs linear measurements, 0.989 (95% CI 0.975–0.995) vs 0.946 (95% CI 0.880–0.976), *p* = 0.0045. The intra-observer %SDD was similar for volumetric and linear measurements, 9.9% vs 11.8%. However, the inter-observer %SDD was greater for volumetric than linear measurements, 20.1% vs 10.6%. Following oblique reformatting to correct tumour angulation, the mean increase in size was 1.14 mm (*p* = 0.04).

**Conclusion:**

Semi-automated volumetric measurements are more repeatable than linear measurements when measuring small VS and should be considered for use in clinical practice. Oblique orientation of VS may contribute to linear measurement error.

## Introduction

When small or intracannalicular vestibular schwannomas (VS) are observed, around two-thirds may remain stable on serial imaging [1, 2]. For patients with growing tumours, active treatment is offered in the form of radiotherapy or surgery. The ability to accurately determine whether VS have grown or are stable is essential for decision-making as well as determining efficacy of treatment.

Despite the improved quality and sensitivity of MRI scanning over the last 3 decades, the potential for observer variation when using linear measurements is well recognized [3, 4]. It has been suggested that growth should be defined as an increase in linear dimension of 2 mm or more, since a change in size of less than 2 mm could be due to measurement error [3–5]. As a result, volumetric measurements have been considered as a potentially more accurate measurement of VS size [6–16].

The benefit of volumetric measurements of VS has been particularly highlighted within the setting of neurofibromatosis type 2 (NF2) [17–19]. This is largely due to the introduction of novel biological therapies such as bevacizumab where reliable monitoring of response is key [6, 19, 20]. In addition, patients with NF2 have larger, more irregular, or lobular tumours which are more difficult to measure accurately with linear dimensions [8].

The majority of previous studies showing the benefits of volumetric over linear measurements of VS have used the manual segmentation or Cavilieri method where the tumour is manually outlined on each MRI slice and the area multiplied by slice thickness [10, 14, 16, 21]. This is a time-consuming technique taking 15–25 min per tumour measurement [10, 14], making it impractical for routine clinical practice. Semi-automated volume segmentation tools (automated tumour outline with manual checking and automated propagation through MRI slices) have become available but are not widely used in clinical practice.

We hypothesized that volumetric measurement of small VS may not provide a significant advantage over conventional linear measurements given that these tumours often have a more uniform ellipsoid shape with a greater intracannalicular proportion than larger tumours [13]. The previous volumetric vs linear comparison studies have included patients with all tumour sizes and patients with NF2 [6, 8, 18, 22, 23] or excluded patients with purely intracanalicular tumours [3]. However, sporadic (non-NF2 related) tumours comprise 95% of all newly diagnosed VS and of these, more than half are intracanalicular or small (less than 15 mm maximum intracranial dimension) and are most likely to be managed initially with observation and serial imaging [24].

This study aimed to determine if semi-automated volume segmentation was more precise than the conventional linear methods for measuring sporadic small and intracanalicular VS. A further aim was to determine if oblique orientation of the long axis of the tumour could contribute to linear measurement error, and if so, whether this could be addressed by oblique reformatting to improve linear measurement accuracy.

## Methods

A departmental database was screened to identify all patients with a unilateral intracanalicular or small sporadic VS imaged with T1-weighted gadolinium contrast-enhanced MRI acquired with 1 mm slice thickness within the last 3 years. These imaging requirements were selected as optimal for volumetric analysis and oblique reformatting but were only available in patients who had undergone 3D image acquisition with isotropic voxels for stereotactic radiotherapy planning. Patients with NF2, aged under 18 or tumours larger than 15 mm maximum intracranial dimension were excluded. Tumours with maximum intracranial dimension less than 15 mm are defined as ‘small’ in line with the British National Vestibular Schwannoma Audit, however, for the purpose of this study, tumour measurements also included the intrameatal (intratemporal component) and are presented as maximum axial dimension.

Two observers (one neuroradiologist and one neurotologist) independently measured each tumour using both linear and semi-automated volumetric techniques. Both observers were blinded to any previous measured values and repeated measurements were made to provide intra and inter-observer repeatability comparisons.

**Fig. 1:**
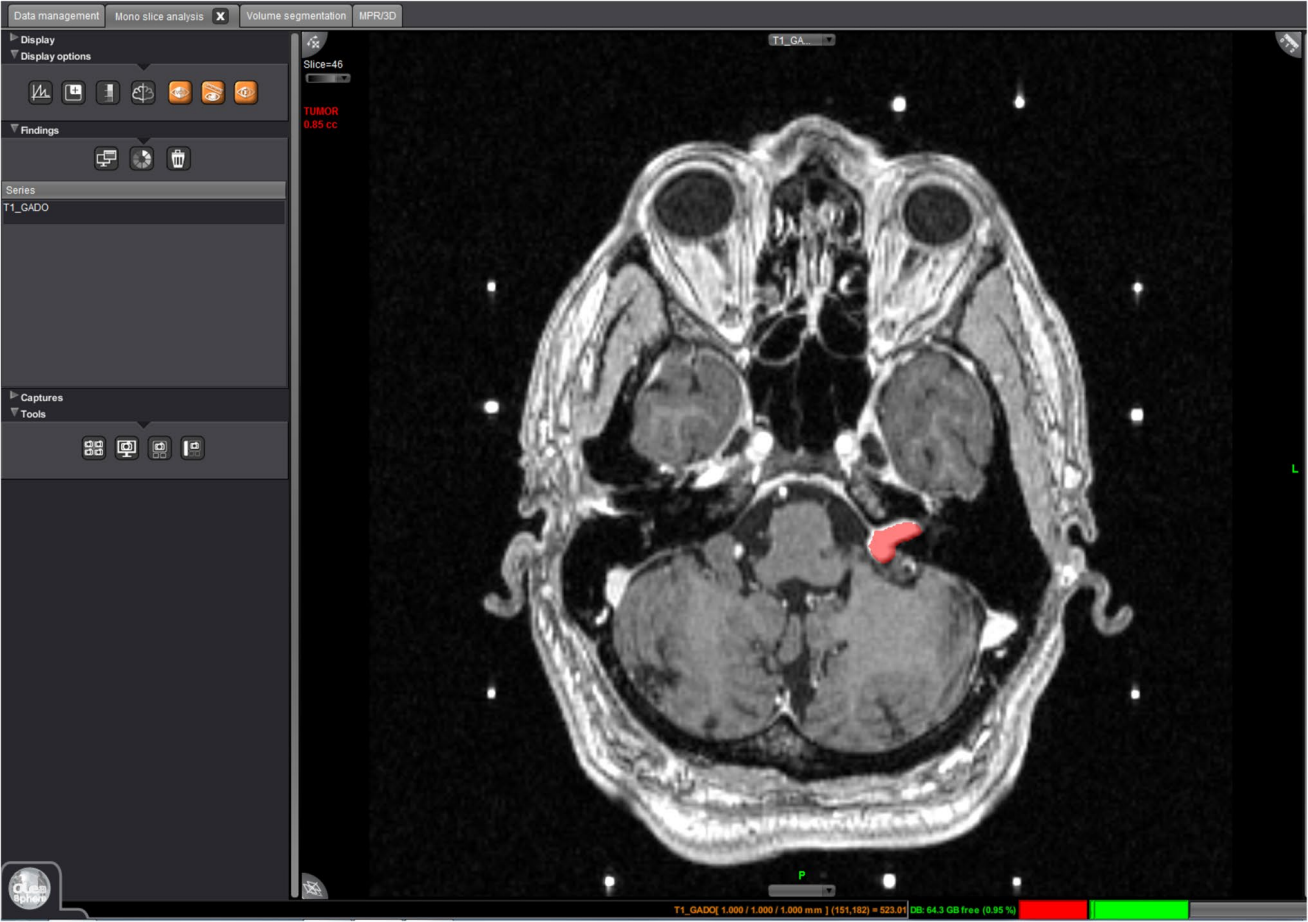
Image of volumetric measurement being made with the Olea Sphere programme. The left VS is highlighted as the ROI (*VS* vestibular schwannoma, *ROI* region of interest)

Linear measurements were made in the axial plane using a digital caliper on a GE Advantage Workstation version 4.5 (GE Healthcare, Waukesha, USA), taking the maximum axial dimension including the intracanalicular component. Volumetric measurements were made using Olea Sphere^®^ Version 2.3 (Olea Medical^®^, La Ciotat, France), a post-processing application with volumetric analysis modules, available commercially for clinical use. The ‘magic wand’ function was used to select the tumour and to create a region of interest (ROI). This uses a ‘region-growing tool’, selecting a growing volume whose voxel values are close to the initially selected voxel, propagated to all other imaging slices to cover the entire tumour (Fig. 1).

### Oblique reformatting

**Fig. 2:**
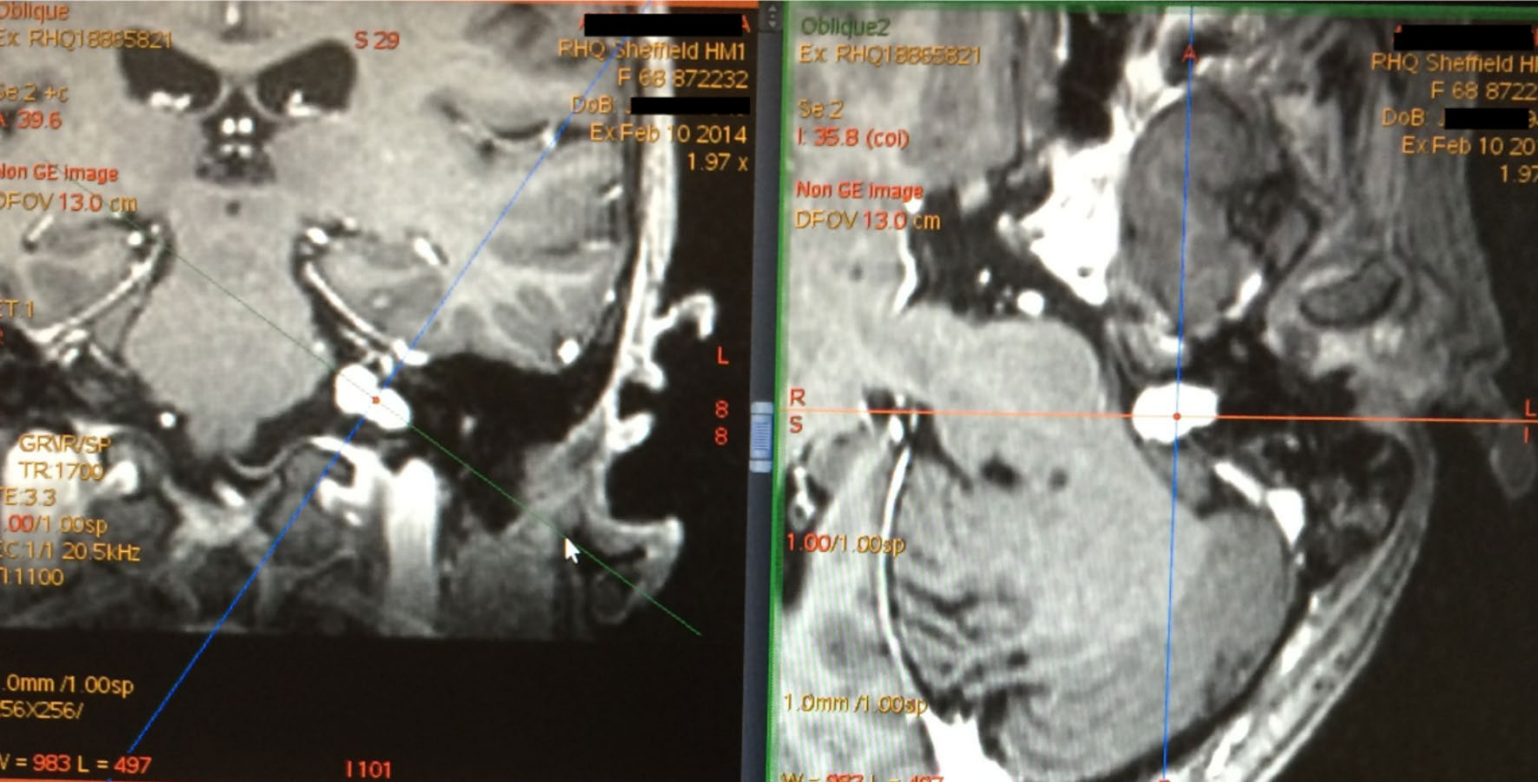
MRI of left VS showing long axis of tumour at 40° angle to the horizontal when viewed in coronal plane (left image), with the obliquely reformatted axial image displayed on the right

All tumours were also assessed in the coronal plane and the angle of the tumour long axis with respect to the axial plane was measured. Where this was greater than an arbitrary threshold of 30 degrees, the oblique reformatting tool on the GE Advantage Workstation was used to produce axial slices transecting the tumour through its long axis (Fig. 2). The maximum linear dimension was compared when measured before and after oblique reformatting.

### Statistical analysis

Data analysis was performed using SPSS Version 23 (SPSS, Chicago, Illinois, USA). Intraclass correlation coefficients (ICC) were calculated for intra and inter-observer paired measurements to compare measurement techniques. A Fisher *r* to *z* calculation was performed to test for significant differences between ICC values. Since correlation measurement data may be highly correlated whilst having poor agreement [25], Bland–Altman plots have been used to graphically demonstrate intra and inter-observer agreement for both measurement techniques. Upper and lower limits of agreement (LoA) were calculated for each set of measurements and incorporated into the Bland–Altman Plots [26, 27].

The Repeatability Coefficient (RC) is the difference which will be exceeded by only 5% of pairs of measurements on the same tumour [25] (RC = Standard deviation of the differences between data pairs × 1.96). To allow for more direct comparison between linear and volumetric measurements values, RC was also converted into proportions given as the relative smallest detectable difference [%SDD = (RC/mean tumour size) × 100].

## Results

Twenty-four cases were included in the study. The mean VS size as determined by maximum axial linear dimension was 14.6 mm (SD = 2.5 mm) and mean tumour volume was 546 mm^3^ (SD = 383mm^3^).

### Linear vs semi-automated volumetric measurements

**Fig. 3:**
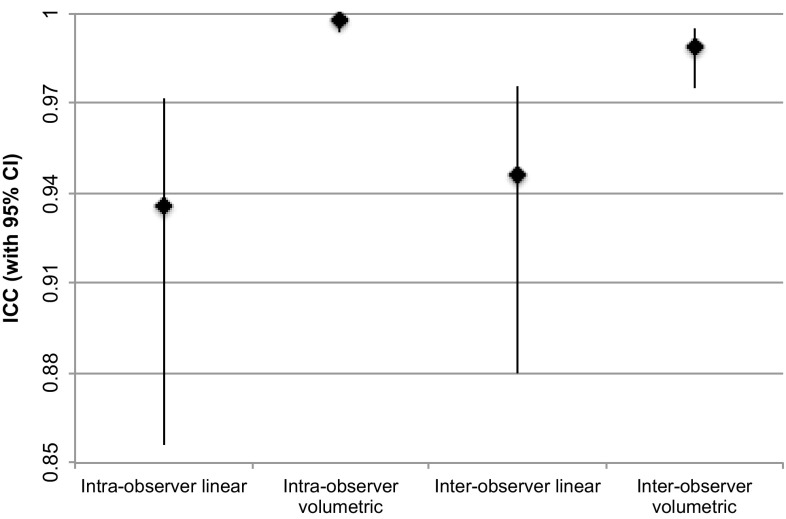
Intra and inter-observer ICC (denoted by filled diamond) for linear and semi-automated volumetric measurements with 95% confidence intervals displayed as high-low lines

Intraclass correlation coefficients (ICC) were excellent for all paired measurements (> 0.9). Table 1 shows that the ICC were significantly higher for semi-automated volumetric measurements than for linear measurements, especially when comparing intra-observer measurements (*p* < 0.0001). Figure 3 plots all four ICC with corresponding 95% confidence intervals (CI) also demonstrating the narrower range of 95%CI for semi-automated volumetric measurements. Although this figure shows the 95% CI for inter-observer linear and semi-automated volumetric ICC are minimally overlapping, a Fishers *r* to *z* transformation confirms the difference between the two ICCs is still significant (*p* = 0.0045).

**Table 1:** demonstrates the intraclass correlation coefficient (ICC) for each set of paired measurements where 0 is no correlation and 1 is perfect correlation

	Intra-observer maximum linear dimension	Inter-observer maximum linear dimension	Intra-observer semi-automated volumetric	Inter-observer semi-automated volumetric
Intraclass correlation coefficient (ICC) (95% confidence intervals)	0.936 (0.856–0.972)	0.946 (0.880–0.976)	0.998 (0.994–0.999)	0.989 (0.975–0.995)
Repeatability coefficient (RC)	1.73 mm	1.65 mm	54 mm^3^	110 mm^3^
Relative smallest detectable difference (%SDD)	11.8%	10.6%	9.9%	20.1%

The repeatability coefficient (RC) and relative smallest detectable differences (%SDD) are also displayed

**Fig. 4:**
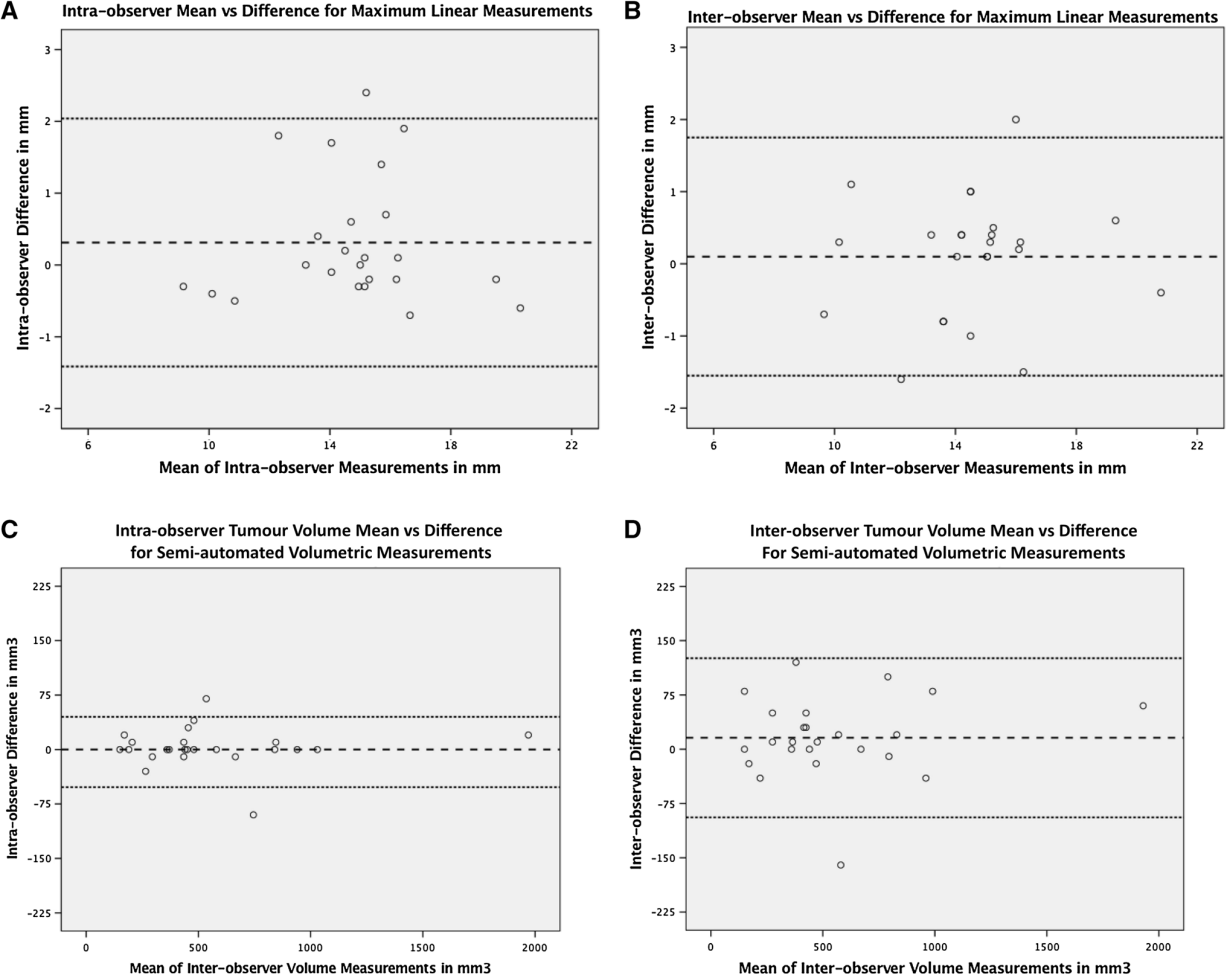
**a**–**d** Display standard Bland–Altman plots for all four paired measurement sets to show levels of intra and inter-observer agreement for linear and semi-automated volumetric measurement techniques. These plot the difference between the values against the mean for each pair of measurements. The dashed line represents the overall mean of the differences between sets of measurements. The dotted lines are calculated as ± 1.96 × SD and represent the upper and lower limits of agreement

In addition to a comparison of correlation, levels of agreement are displayed in Fig. 4a–d in standard Bland–Altman plots. Equal distribution of points plotted above and below the mean of the difference (dashed line) confirms no systematic difference between the first and second sets of measurements of the same tumour (*p* > 0.05). Furthermore, there does not appear to be a relation between magnitude of error and size of tumour. This is confirmed with poor correlation coefficients between tumour size and intra/inter-observer measurement difference (< 0.1 for all four data sets).

Repeatability coefficients (RC) were less than 2 mm for both intra and inter-observer linear measurements (1.73 and 1.65 mm). Linear measurement relative smallest detectable difference (%SDD) was similar for intra vs inter-observer measurements (11.8 and 10.6% respectively). However, volumetric intra-observer %SDD was half that of the inter-observer measurement (9.9 vs 20.1%).

### Linear measurement following oblique reformatting

In 5 out of 24 cases, review of images in the coronal plane identified the long axis of the tumour to be angulated greater than 30° from the axial plane. Following oblique reformatting, all 5 were deemed to be larger on repeat measurement, with a mean increase in size of 1.14 mm (range 0.2–2.7 mm; *p* = 0.04).

## Discussion

The improvement in the observer agreement using the semi-automated volumetric technique suggests that this method is more *precise* than the conventional linear method. In the absence of comparing both measurement techniques against a gold standard, such as comparison to a phantom tumour of known size, it is not possible to know how *accurate* each technique is. However, the key information required when measuring VS in clinical practice is whether or not there is change in size. A measurement technique that demonstrates better observer agreement will likely be both sensitive to, and more reliable at, correctly detecting growth.

Although this study directly compares correlation coefficients between linear and semi-automated volumetric measurements, this comparison is potentially flawed, as stated previously, as measurement data may be highly correlated with poor agreement. Bland–Altman plots have, therefore, been constructed and repeatability coefficients (RC) calculated. The RC is the smallest true change in tumour size that can be reliably detected from two separate measurements. The previous studies have used the RC to help quantity a minimum increase in size that should be regarded as growth [3, 4, 8, 10, 13, 14].

Unfortunately, it is not possible to compare the RC of linear and volumetric measurement techniques due to differing units (e.g., 1.73 mm vs 54 mm^3^). In an attempt to reconcile this and allow more direct comparison, the relative smallest detectable difference values are presented (%SDD) [3, 8, 13].

It is interesting to note that despite the higher inter-observer ICC of semi-automated volumetric technique, the %SDD is twice that of linear measurements (10.6 vs 20.1%). This could be misleading in suggesting that the volumetric technique has a greater margin of error when detecting change in size of VS. However, these conflicting results may be explained by geometric differences between one- and three-dimensional measurements. For example, if an ellipsoid VS of dimensions (20 × 10 × 10 mm) increases in size by 10% in all directions, its maximal linear dimension will have increased from 20 to 22 mm, whereas the increase in volume will be from 1047 to 1394 mm^3^, an increase of 32%. Therefore, depending on the geometric shape of the tumour and in which dimension it increases, the volumetric technique (even with a larger %SDD of 20%) is likely to be more sensitive at detecting growth than the linear method.

The difference between the %SDD for intra vs inter-observer measurements made with the semi-automated volume segmentation tool (9.9 vs 20.1%) highlights that the semi-automated process is not without risk of human error. In this study, variation in the initial selection of the region of interest, manual editing out of highlighted peritumoural vessels, and the need to manually highlight areas of hypointensity not included in the automated segmentation were all thought to contribute to inter-observer variation.

This study does, however, have limitations that should be considered when interpreting the results. The imaging protocol used (T1-weighted gadolinium contrast-enhanced MRI acquired with 1 mm slice thickness) is not standard in many units, and as such, the results may not be generalizable for use with other imaging protocols, especially in units where balanced Steady-State Free Precession (bSSFP) sequences are used in place of contrast-enhanced scans. This study tests just one commercially available semi-automated volume segmentation tool; the results may not necessarily be representative of other similar post-processing applications. Maximum axial tumour dimension was used as the linear measurement method. Although other methods are common, there is some evidence that this is the optimal dimension for reliability [3].

When considering linear measurement error, there are a range of factors that may contribute including differences in image acquisition such as slice thickness, change in head position, degree of contrast enhancement, and adjustment of the tumour image with windowing [28, 29]. To our knowledge, this is the first study to demonstrate that oblique reformatting may correct linear measurement error due to oblique tumour orientation. The decision to only reformat tumours that were obliquely orientated greater than 30° was based on a pragmatic approach more reflective of clinical practice where reformatting would only be undertaken when the long axis of the tumour was clearly in an oblique lie to the plane of measurement. A limitation of this technique is that it requires image slices acquired with volumetric isotropic 1 mm voxels, which are not necessarily available in routine practice.

### Comparison with other studies

Many of the previously reported studies use a manual segmentation volume measurement technique which is impractical for use in daily clinical practice [14, 16, 21, 30]. Some studies have used a semi-automated volume segmentation tool which uses an ‘active contouring’ algorithm to outline the tumour on each slice; each slice area is then automatically summated and multiplied by slice thickness to provide a volume [13, 18]. The semi-automated volume segmentation tool used in the present study (Olea Sphere) utilizes a ‘region-growing’ algorithm to outline the tumour on a single axial slice and automatically propagates the region of interest through adjacent slices. The estimated time taken to perform a single semi-automated volumetric measurement using Olea Sphere was 60–90 s for both observers. This is considerably faster than the previous reports of semi-automated volume segmentation (3 and 4–7 min) [13, 16] and manual segmentation (15–25 min) [10, 14]. As well as reducing the time required, the increased automation of current tools has the potential to standardize some of the measurement process which may also improve repeatability.

In this study, the inter-observer %SDD was 20.1% for semi-automated volumetric measurements which is comparable to other similar studies [8, 13–15]. As suggested in the previous reports and supported by our results, an increase in volume of at least 20% is required to be considered evidence of tumour growth.

The linear intra-observer and inter-observer RC were better than the previous reports [3, 4, 13]. This may be explained by the 1 mm image slice thickness used in this study allowing more accurate measurements. In addition, in this study, both observers work within the same MDT, and this may have resulted in standardization of practice such as windowing and boundary edge judgment.

### Implications for clinical practice

It is clear from this study, and others than volumetric measurements are more repeatable, precise and will, therefore, detect true change in VS size better (and therefore earlier) than the conventional linear measurements. Whilst manual segmentation was impractical and largely limited to use in research, the advent of faster more automated volume analysis tools provides the opportunity for volumetric measurements to be incorporated into routine clinical practice.

Clinical research examining efficacy of treatments for VS should regard volumetric measurements as essential, given that the increased precision and sensitivity will improve the power of any study often reducing the sample size required. However, it remains unclear how the routine use of volumetric measurements with improved sensitivity would affect patient outcomes.

Our understanding of the natural history of VS is based on studies of which almost all used linear measurements [1, 2, 5, 31]. It is conceivable that management decisions based on evidence of growth from a much more sensitive measurement technique could lead to a larger numbers of patients receiving active treatment. However, the earlier detection and treatment of VS may not translate into improved patient outcomes, since a small change in volume is unlikely to result in worse outcomes in terms of tumour control after stereotactic radiosurgery [32]. Moreover, it is recognized that tumours have a variable growth behavior and a minority may grow a small amount then cease to grow further. This raises the possibility that earlier treatment could result in a minority potentially receiving treatment unnecessarily. Further research should aim to answer these questions.

If semi-automated volume segmentation applications are to be used routinely, other factors such as usability and cost should be considered and compared between available tools. For units continuing with linear measurements, awareness of oblique orientation and reformatting to correct this may help to reduce linear measurement error.

## Conclusion

This study demonstrates higher repeatability of semi-automated volume segmentation measurement technique over standard linear method for measurement of sporadic intracanalicular and small VS. In line with the previous research, our results support a linear measurement growth criteria of > 2 mm and a volumetric increase > 20%.

If semi-automated volumetric analysis tools are to be used more routinely, there is a requirement to compare and evaluate the various available post-processing applications in an effort to identify the optimal algorithms for use with VS. While linear measurement method remains current practice in most units, the orientation of VS with respect to the horizontal plane should be noted, and if over 30°, oblique reformatting should be considered to reduce linear measurement error.
